# Predictive value of expression of p16^INK4A^, retinoblastoma and p53 proteins for the prognosis of non-small-cell lung cancers

**DOI:** 10.1038/sj.bjc.6690750

**Published:** 1999-10

**Authors:** F Hommura, H Dosaka-Akita, I Kinoshita, T Mishina, H Hiroumi, S Ogura, H Katoh, Y Kawakami

**Affiliations:** First Department of Medicine, Hokkaido University School of Medicine, North 15, West 7 Kita-ku, Sapporo, 060-8638, Japan; Second Department of Surgery, Hokkaido University School of Medicine, Sapporo, 060-8638, Japan

**Keywords:** cell cycle regulator, G1 checkpoint, clinical outcome

## Abstract

The predictive value of expression of p16^INK4A^, retinoblastoma (Rb) and p53 proteins for prognosis was evaluated in 76 patients with non-small-cell lung cancers (NSCLCs) that were potentially curatively resected between 1990 and 1995, using the results of immunostaining analyses of these proteins as reported in our previous study (Kinoshita et al, 1996). Of these NSCLCs, 22 (29%) lacked p16 protein expression and eight (11%) Rb protein, while 30 (39%) showed positive (altered) p53 protein expression. Survival of patients with p16-negative tumours was not significantly different from that of patients with p16-positive tumours (5-year survival rates 67% and 72% respectively, *P* = 0.8), nor was survival of patients with Rb-negative tumours significantly different from that of patients with Rb-positive tumours (5-year survival rates 42% and 69% respectively, *P* = 0.9). Moreover, survival of patients with p16/Rb-negative (either p16- or Rb-negative) tumours was not significantly different from that of patients with p16/Rb-positive (both p16- and Rb-positive) tumours (5-year survival rates 67% and 68% respectively, *P* = 0.7). In contrast, survival of patients with p53-positive (altered) tumours tended to be shorter than that of patients with p53-negative (unaltered) tumours (5-year survival rates 56% and 78% respectively, *P* = 0.06). In univariate analysis of potential prognostic factors, p16, Rb and p16/Rb proteins were not significant prognostic factors in the present cohort of potentially curatively resected NSCLCs. Altered p53 protein status tended to be a negative prognostic factor (*P* = 0.06 by the univariate analysis). These results indicate that loss of p16 protein alone, or in combination with loss of Rb protein, does not predict the clinical outcome of patients with resected NSCLCs. © 1999 Cancer Research Campaign


*British Journal of Cancer* (1999) **81**(4), 696–701
© 1999 Cancer Research Campaign
Article no. bjoc.1999.0750

### Predictive value of expression of p16$^{INK4A}$, retinoblastoma and p53 proteins for the prognosis of non-small-cell lung cancers

**F Hommura[1], H Dosaka-Akita[1], I Kinoshita[1], T Mishina[1], H Hiroumi[1], S Ogura[1], H Katoh[2] and Y Kawakami[1]**

[1]First Department of Medicine, Hokkaido University School of Medicine, North 15, West 7, Kita-ku, Sapporo, 060-8638, Japan; [2]Second Department of Surgery, Hokkaido University School of Medicine, Sapporo, 060-8638, Japan

**Summary** The predictive value of expression of p16$^{INK4A}$, retinoblastoma (Rb) and p53 proteins for prognosis was evaluated in 76 patients with non-small-cell lung cancers (NSCLCs) that were potentially curatively resected between 1990 and 1995, using the results of immunostaining analyses of these proteins as reported in our previous study (Kinoshita et al, 1996). Of these NSCLCs, 22 (29%) lacked p16 protein expression and eight (11%) Rb protein, while 30 (39%) showed positive (altered) p53 protein expression. Survival of patients with p16-negative tumours was not significantly different from that of patients with p16-positive tumours (5-year survival rates 67% and 72% respectively, $P = 0.8$), nor was survival of patients with Rb-negative tumours significantly different from that of patients with Rb-positive tumours (5-year survival rates 42% and 69% respectively, $P = 0.9$). Moreover, survival of patients with p16/Rb-negative (either p16- or Rb-negative) tumours was not significantly different from that of patients with p16/Rb-positive (both p16- and Rb-positive) tumours (5-year survival rates 67% and 68% respectively, $P = 0.7$). In contrast, survival of patients with p53-positive (altered) tumours tended to be shorter than that of patients with p53-negative (unaltered) tumours (5-year survival rates 56% and 78% respectively, $P = 0.06$). In univariate analysis of potential prognostic factors, p16, Rb and p16/Rb proteins were not significant prognostic factors in the present cohort of potentially curatively resected NSCLCs. Altered p53 protein status tended to be a negative prognostic factor ($P = 0.06$ by the univariate analysis). These results indicate that loss of p16 protein alone, or in combination with loss of Rb protein, does not predict the clinical outcome of patients with resected NSCLCs. © 1999 Cancer Research Campaign

**Keywords:** cell cycle regulator; G1 checkpoint; clinical outcome p16 Protein, the product of the *CDKN2/MTS1* gene, inhibits cyclin-dependent kinase (cdk) 4- and cdk6-mediated phosphorylation of retinoblastoma (Rb) protein (Serrano et al, 1993; Hannon and Beach, 1994). Both p16 and Rb proteins, which, like p53 protein, regulate cell cycle progression at the G1 checkpoint, function in a single regulatory pathway of the cell cycle and tumour suppression (Serrano et al, 1993; Lukas et al, 1995), a conclusion supported by the findings of inverse correlations between alteration of both proteins in several types of cancer, including non-small-cell lung cancers (NSCLCs) (Okamoto et al, 1994; Otterson et al, 1994; Tam et al, 1994; Geradts et al, 1995; Kelley et al, 1995; Shapiro et al, 1995).

It has been shown in NSCLCs that homozygous deletion and DNA methylation are common mechanisms of *CDKN2/MTS1* gene inactivation (Cairns et al, 1994; Hayashi et al, 1994; Nakagawa et al, 1995; Okamoto et al, 1995; Otterson et al, 1995; Shapiro et al, 1995) and that mutations of the gene are mostly restricted to metastatic sites (Nakagawa et al, 1995; Okamoto et al, 1995). In a previous study (Kinoshita et al, 1996), we demonstrated that immunohistochemical analysis of p16 protein in tumour cells, using admixed stromal cells retaining normal p16 expression as an internal control, was a sensitive and suitable method to screen for *CDKN2* gene inactivation in NSCLCs. Similarly, most *Rb* gene alterations result in the loss of Rb protein expression or in a truncated Rb protein, which does not enter the nucleus. Heterogeneous positive nuclear Rb immunostaining is, in general, indicative of normal Rb function, whereas negative intranuclear Rb immunostaining in all tumour cells reflects functional loss of the *Rb* gene (Xu et al, 1991*a*, 1991*b*). Moreover, to date, the presence or absence of normal Rb protein expression determined by immunohistochemistry has been found to be the most sensitive and specific method to examine Rb status in a given tumour (Zhang et al, 1994).

Using the same cohort as the present study, we have previously reported the reciprocal losses of p16 and Rb proteins and the potential synergistic effect of the altered p16/Rb (p16 protein in combination with Rb protein) pathway with altered p53 protein on the proliferative activity in NSCLCs (Kinoshita et al, 1996). We have also reported that Rb protein status alone was not a predictor of survival and prognosis in a different cohort of NSCLC patients (Dosaka-Akita et al, 1997). The prognostic value of p16 alterations in NSCLCs has been reported only in a few studies (Kelley et al, 1995; Kratzke et al, 1996; Taga et al, 1997), and is still controversial. Moreover, no studies have clearly shown the importance of p16/Rb protein status for the clinical outcome of patients with NSCLCs, although both p16 and Rb proteins are involved in a single regulatory pathway of the cell cycle and tumour suppression, and are reciprocally lost in NSCLCs.

*Received 25 September 1998*
*Revised 8 February 1999*
*Accepted 11 March 1999*

*Correspondence to:* H Dosaka-Akita

**Table 1** p16 and p16/Rb protein expression in 76 resected NSCLCs

| | p16 protein status | | | p16/Rb protein status | | |
|---|---|---|---|---|---|---|
| **Characteristics** | **(+)** | **(–)** | ***P*[a]** | **(+)[b]** | **(–)[c]** | ***P*[a]** |
| | 54 (71%) | 22 (29%) | | 46 (61%) | 30 (39%) | |
| Age (mean ± s.d.) | 62.6 ± 8.5 | 61.9 ± 9.6 | 0.7 | 62.4 ± 8.8 | 62.4 ± 9.0 | 1.0 |
| Gender | | | | | | |
| Male | 40 | 14 | 0.4 | 35 | 19 | 0.2 |
| Female | 14 | 8 | | 11 | 11 | |
| Smoking (pack years) | | | | | | |
| 0–19 | 16 | 9 | 0.3 | 15 | 10 | 0.9 |
| ≥20 | 38 | 13 | | 31 | 20 | |
| Histological type[d] | | | | | | |
| Squamous | 18 | 3 | 0.01 | 16 | 5 | 0.06 |
| Non-squamous | 36 | 19 | | 30 | 25 | |
| pStage | | | | | | |
| I | 34 | 17 | 0.5 | 30 | 21 | 0.9 |
| II | 7 | 2 | | 6 | 3 | |
| IIIa | 13 | 3 | | 10 | 6 | |
| Chemotherapy[e] | | | | | | |
| (–) | 36 | 16 | 0.6 | 32 | 20 | 0.8 |
| (+) | 18 | 6 | | 14 | 10 | |
| Rb | | | | | | |
| (+) | 46 | 22 | 0.06 | | | |
| (–) | 8 | 0 | | | | |
| p53 | | | | | | |
| (–) | 35 | 11 | 0.2 | 32 | 14 | 0.046 |
| (+) | 19 | 11 | | 14 | 16 | |

[a]The associations between loss of p16 and p16/Rb proteins and characteristics of patients except for age were analysed by $\chi^2$ test (age analysed by Student's *t*-test). [b](+), retaining both p16 and Rb proteins. [c](–), lacking either p16 or Rb protein. [d]Squamous, squamous cell carcinoma; Non-squamous, non-squamous cell carcinoma, including adenocarcinoma and large-cell carcinoma. [e]Chemotherapy, post-surgical chemotherapy.

In the present study, we investigated the prognostic significance of p16, Rb and p16/Rb protein status determined by immunohistochemistry in patients with resected NSCLCs.

### MATERIALS AND METHODS

### Patients and survival data

Of 111 NSCLCs studied in our previous report (Kinoshita et al, 1996), 92 tumours were resected with curative intent. Survival was analysed in the present study for the 76 patients who met the following criteria: (1) survived for more than 3 months after surgery; (2) did not die of causes other than lung cancer within 5 years after surgery; and (3) were followed for more than 2 years after surgery (for patients who remained alive). Twelve patients who did not meet the above criteria were excluded from the present study. Of the 12 patients, two died within 3 months after surgical resection, five died of causes other than lung cancer within 5 years after surgery, and five were followed up for no more than 2 years after surgery. Four patients for whom no survival records after surgery were obtained were also excluded from the present study. Of the 76 patients analysed for survival, 54 were men and 22 were women. Age and tobacco consumption at time of surgery (mean ± standard deviation (s.d.) were 62.4 ± 8.8 years and 38.8 ± 33.5 pack years respectively. Fifty-eight were still alive after surgery with a median follow-up of 51 months (range 25–76). According to the 1981 WHO classification (WHO, 1982), tumour specimens were histopathologically diagnosed as adenocarcinoma ($n$ = 50), squamous cell carcinoma ($n$ = 21) and large-cell carcinoma ($n$ = 5). They represented 51 stage I, nine stage II and 16 stage IIIa tumours. The post-surgical pathological TNM stage (pTNM) was determined according to the guidelines of the American Joint Committee on Cancer (Beahrs et al, 1992). Twenty-four patients received combination chemotherapy as post-surgical treatment. Radiation therapy was not performed before or after surgery for any patient. Because all the patients enrolled in the current study were coded, they could not be individually identified.

### Immunohistochemistry for p16, Rb, and p53 proteins

For the p16, Rb and p53 protein staining, the slides and results that were previously reported (Kinoshita et al, 1996) were used for the present study. The methods for staining of the p16, Rb, and p53 nuclear proteins of tumour cells have been described (Kinoshita et al, 1996). Briefly, tumours from the primary NSCLCs were snap-frozen in liquid nitrogen and stored at –80°C until use. Antibodies used for immunohistochemistry of p16, Rb, and p53 proteins were rabbit polyclonal anti-entire-human p16 antibodies (PharMingen, San Diego, CA, USA), and mouse monoclonal antibodies PMG3-245 (PharMingen) and PAb1801 (Oncogene Science, Manhasset, NY, USA) respectively. Immunostaining was performed by the biotin-streptavidin immunoperoxidase method with 3,3′-diaminobenzidine as a chromogen (SAB-PO kit, Nichirei, Tokyo, Japan). Methyl green was used for counterstaining.

The criteria for the evaluation of p16, Rb, and p53 proteins have been described previously (Xu et al, 1991; Dosaka-Akita et al, 1994, 1997; Kinoshita et al, 1996). Briefly, tumours were scored as p16-negative (p16–) or Rb-negative (Rb–) if all malignant cells

© 1999 Cancer Research Campaign 

**Table 2** Univariate and multivariate analyses of potential prognostic factors in resected NSCLCs

| Characteristics | Hazards ratio | 95% confidence interval | $P^a$ |
|---|---|---|---|
| A. Univariate analysis | | | |
| Age | 0.99 | 0.95–1.03 | 0.6 |
| Gender | 2.13 | 0.73–6.24 | 0.2 |
| Histological type[b] | 1.34 | 0.58–3.14 | 0.5 |
| pStage | 1.71 | 1.13–2.58 | 0.01 |
| Chemotherapy | 0.76 | 0.33–1.75 | 0.5 |
| p16 | 0.88 | 0.35–2.25 | 0.8 |
| Rb | 0.88 | 0.21–3.79 | 0.9 |
| p16/Rb | 0.86 | 0.36–2.03 | 0.7 |
| p53 | 2.15 | 0.96–4.81 | 0.06 |
| B. Multivariate analysis | | | |
| pStage | 2.56 | 1.03–6.41 | 0.03 |
| p53 | 1.92 | 0.85–4.33 | 0.1 |

[a]Analysed by Cox's proportional hazards general linear model. [b]Squamous cell carcinoma vs non-squamous cell carcinoma.

had no nuclear staining, but surrounding normal stromal cells showed adequate nuclear staining for p16 or Rb protein, respectively, as positive internal controls. Tumours were regarded as p16-positive (p16+) or Rb-positive (Rb+) if any malignant cell had nuclear staining for p16 or Rb protein respectively. Tumours were considered to be p53 alteration-positive (altered) (p53+) and thus to contain putative p53 gene mutations when more than 10% of tumour cells showed nuclear staining for p53 protein, and were scored as p53 alteration-negative (unaltered) (p53–) in the other cases.

### Statistical analysis

The associations between losses of p16, Rb, p16/Rb and p53 proteins and various characteristics of patients and tumours were analysed by the $\chi^2$ test. Those between losses of p16, Rb, p16/Rb and p53 proteins and age were analysed by Student's *t*-test. Survival curves of patients with tumours having different p16 and/or Rb protein status were estimated using the Kaplan–Meier method (Kaplan and Meier, 1958) and differences in survival distributions were evaluated by the generalized Wilcoxon test (Gehan, 1965). Prognostic values of p16, Rb, p16/Rb and p53 proteins were estimated by univariate and multivariate analyses using Cox's proportional hazards general linear model (Harrell, 1986). The significance level chosen was $P < 0.05$, and all tests were two-sided. These computations were performed with the SAS program package (Harrell, 1986).

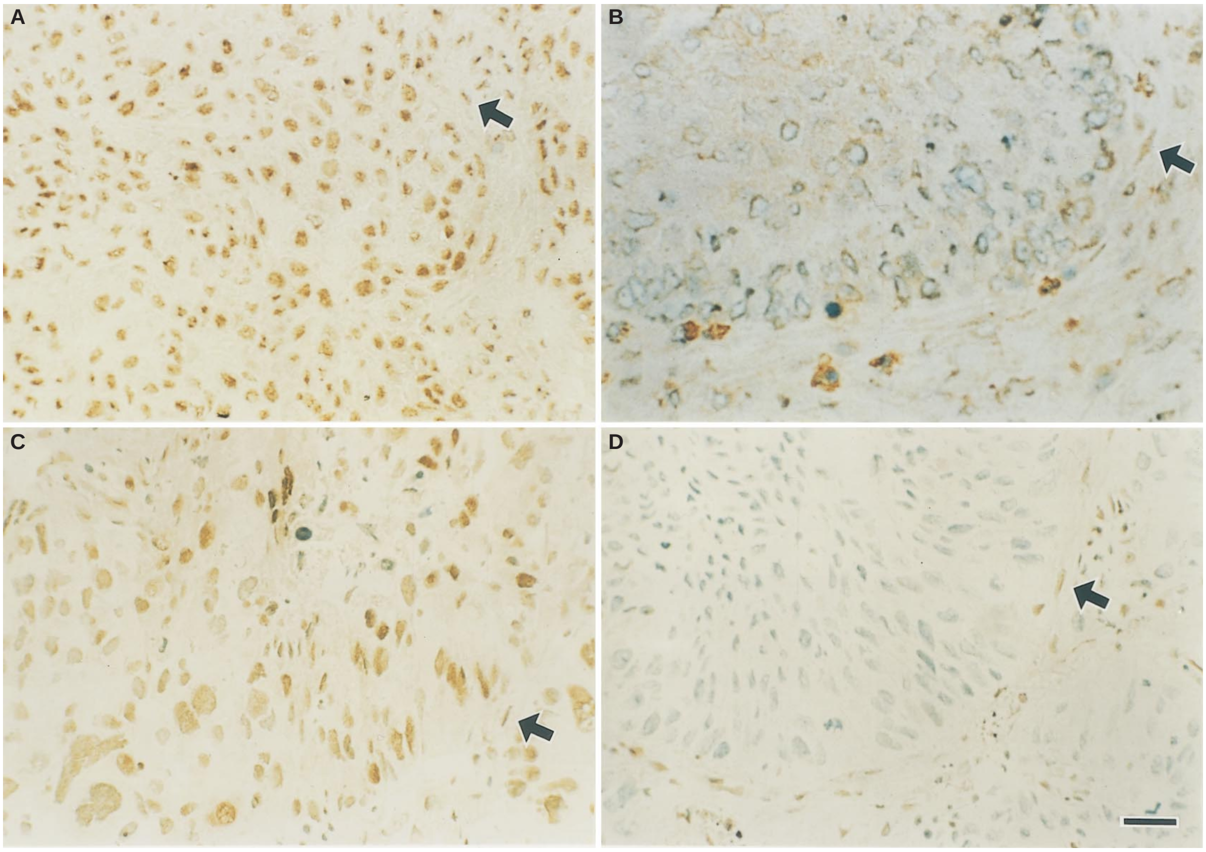

**Figure 1** Immunostaining patterns for p16 (**A, B**) and Rb (**C, D**) proteins in primary NSCLCs. Representative p16+ adenocarcinoma shows strong and homogeneous nuclear staining of p16 protein in tumour cells (**A**). In contrast, p16– squamous cell carcinoma shows no nuclear staining of p16 protein in tumour cells (**B**). Representative Rb+ squamous cell carcinoma displays intense nuclear staining of Rb protein in tumour cells (**C**). Rb-adenocarcinoma displays no nuclear staining of Rb protein in tumor cells (**D**). Note that admixed stromal cells (arrows) show distinct nuclear staining of p16 and Rb proteins in all specimens, which provide positive internal controls for both proteins. **A–D**, ×400. Scale bar = 20 μm

© 1999 Cancer Research Campaign

### RESULTS

Of 111 NSCLCs analysed in the previous study (Kinoshita et al, 1996), 76 tumours were studied to determine the prognostic importance of p16 protein status alone or in combination with Rb protein status (p16/Rb) in the present study. Typical immunostaining for p16 and Rb proteins in NSCLCs is shown in Figure 1. Twenty-two (29%) NSCLC tumours lacked p16 protein expression, and eight (11%) lacked Rb protein. Thus, 30 (39%) NSCLCs had a loss of either p16 or Rb protein, while 30 (39%) showed positive (altered) p53 protein expression (Table 1).

Survival of patients with p16– and p16+ tumours was very similar, and not significantly different (5-year survival rates 67% and 72% respectively, $P$ = 0.8) (Figure 2A). Survival of patients with Rb– tumours was not significantly different from survival of patients with Rb+ tumours (5-year survival rates 42% and 69% respectively, $P$ = 0.9) (Figure 2B). Finally, survival of patients with tumours lacking either p16 or Rb protein (p16– or Rb–; p16/Rb–) was almost the same as that of patients with tumours retaining both p16 and Rb proteins (p16+ and Rb+; p16/Rb+) (5-year survival rates 67% and 68% respectively, $P$ = 0.7) (Figure 2C). In contrast, survival of patients with p53+ (altered) tumours tended to be shorter than that of patients with p53– (unaltered) tumours (5-year survival rates 56% and 78% respectively, $P$ = 0.06) (Figure 2D). As shown in Table 1, there was no statistically significant difference between the p16+ and p16– groups and between p16/Rb+ and p16/Rb– groups in this cohort with regard to the distributions of other characteristics, including age, gender, smoking, disease stage and post-surgical chemotherapy given. In particular, the data clearly indicated that the two groups were well balanced for disease stage, a previously identified prognostic factor. However, p16 protein was lost more frequently in adenocarcinomas and large-cell carcinomas than in squamous cell carcinomas ($P$ = 0.01). p16/Rb protein status tended to be altered more often in adenocarcinomas and large-cell carcinomas than in squamous cell carcinomas ($P$ = 0.06), but was significantly frequently altered in p53+ tumours compared to p53– tumours ($P$ = 0.046). Inversely, p53– tumours significantly frequently showed unaltered p16/Rb protein status. Statistical significance of potential prognostic factors was determined by univariate analysis (Table 2A). p16, Rb and p16/Rb proteins were not significant prognostic factors, although disease stage was. Altered p53 protein status tended to be a negative prognostic factor ($P$ = 0.06). In the multivariate analysis, pStage was a significant prognostic factor to confirm the reliability of this cohort of potentially curatively resected NSCLCs, but altered p53 protein status was not a significant prognostic factor (Table 2B).

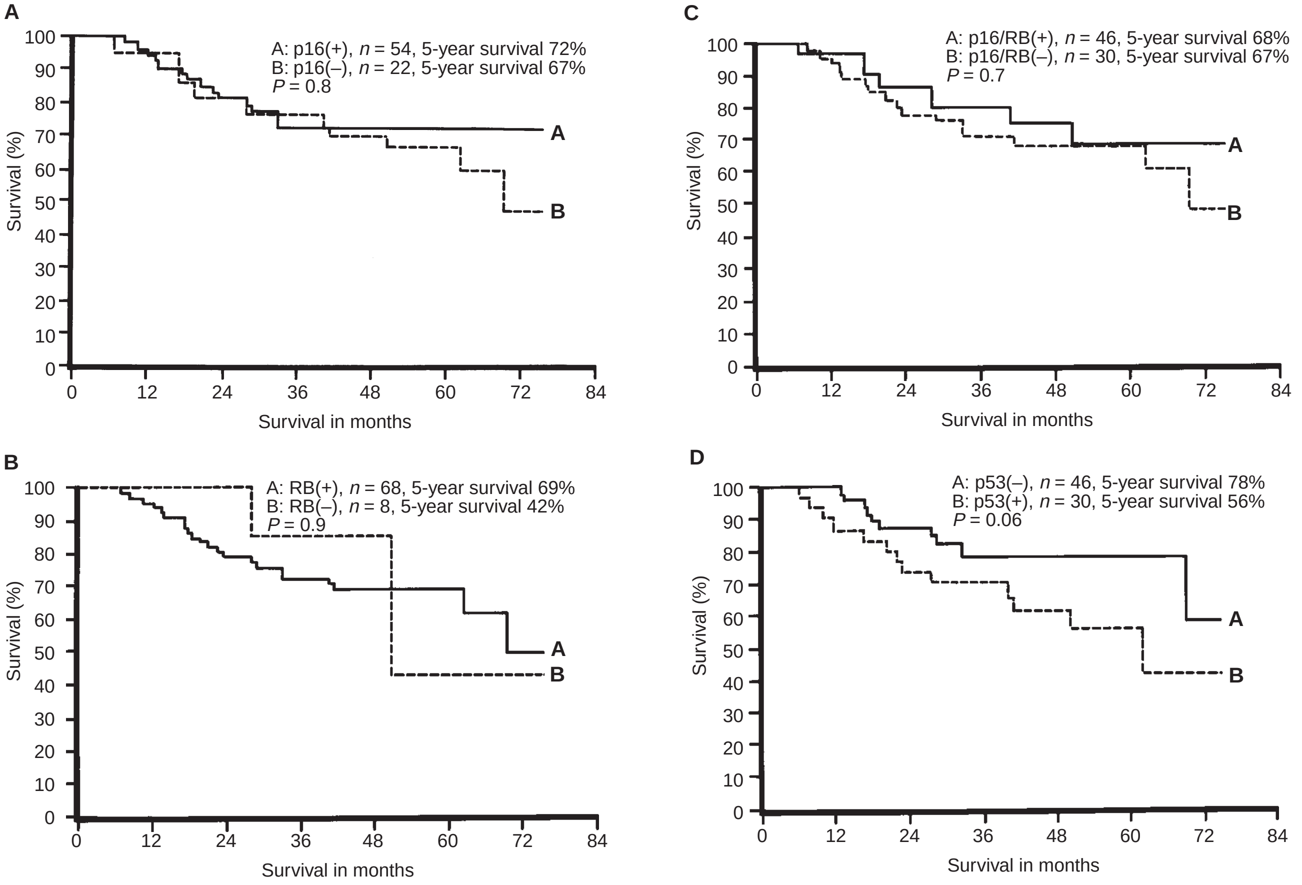

**Figure 2** Kaplan–Meier survival curves of patients with resected NSCLCs. Survival curves of NSCLC patients are stratified by p16 protein (**A**), Rb protein (**B**), p16/Rb protein (p16 protein status in combination with Rb protein status: p16/Rb–, lacking either p16 or Rb protein; p16/Rb+, retaining both p16 and Rb proteins) (**C**), or p53 protein (**D**)

© 1999 Cancer Research Campaign

### DISCUSSION

The present study demonstrated that expression of p16, Rb and p16/Rb in NSCLCs was not associated with survival, and hence of no prognostic value in patients with curatively resected NSCLCs. In contrast, altered p53 protein expression tended to be associated with shorter survival and to be a negative prognostic factor in the present cohort of NSCLCs. p16 and Rb proteins have been shown to be involved in a single regulatory pathway of the cell cycle and tumour suppression (Serrano et al, 1993; Lukas et al, 1995), and to be reciprocally lost in various cancers, including NSCLCs (Okamoto et al, 1994; Otterson et al, 1994; Tam et al, 1994; Geradts et al, 1995; Kelley et al, 1995; Shapiro et al, 1995). Betticher et al (1997) reported that patients with altered Rb expression in their tumours, irrespective of the p16 and cyclin D1 status, tended to have shortened event-free survival for resected NSCLCs. These data suggested that it would be interesting to analyse losses of both p16 and Rb proteins, and to correlate these findings with the clinical outcome of NSCLC patients. In this regard, we investigated prognostic values of losses of both proteins in patients with resected NSCLCs in this study.

Kelley et al (1995) reported that only the cell lines established from stage III and IV NSCLCs had homozygous deletion of the p16 gene, and that the survival of patients having cells with and without the homozygous deletion did not exhibit any significant difference. On the other hand, Kratzke et al (1996) studied p16 protein expression in a cohort of resected NSCLCs by immunohistochemistry using formalin-fixed paraffin-embedded specimens, and reported that loss of p16 protein was associated with shorter survivals, and was a negative prognostic factor. A study by Taga et al (1997) also showed the association of p16 protein loss demonstrated by immunohistochemistry with shorter survivals, but did not show its statistical significance as a prognostic factor. The results of the latter two studies on the relationship between the loss of p16 protein and survival and prognosis of NSCLC patients are not in agreement with ours. This discordance may be attributed to the difference of fixation and preservation of materials; we used fresh, rapidly frozen tissues fixed in methanol, but the other two studies used archived formalin-fixed paraffin-embedded tissues. p16 is considered to be highly susceptible to chemical damage, depending on the condition of the fixation and/or preservation of archived tissues (Geradts et al, 1995). In addition, the antibodies against p16 protein used and the criteria for scoring the presence or absence of p16 protein were different among studies, and may have caused the discordance of the results. Alternatively, the relatively small number of patients analysed in the present study might be the reason that no association of p16 and p16/Rb protein status with clinical outcome was found. However, the present cohort was thought to be suitable for the analysis of clinical outcome in patients with NSCLCs, since disease stage and pN classification (data not shown) were statistically significant prognostic factors associated with survival, as expected. Moreover, survival curves of patients with p16+ and p16– tumours were very similar. Likewise, survival curves of patients with p16/Rb+ and p16/Rb– tumours were very close to each other, suggesting that the lack of association of p16 and p16/Rb protein status with survival was not due to the relatively small number of patients analysed. Taken together, these findings indicate the reliability of the present results on p16 and p16/Rb protein status in relation to survival and prognosis.

For Rb protein, the present results on the relationship between Rb protein status and clinical outcome are consistent with those of our previous study (Dosaka-Akita et al, 1997), in which we reported that loss of Rb protein was not a prognostic factor for overall NSCLCs. Loss of only one or few cell cycle regulators functioning at the G1 checkpoint, such as p16 and Rb proteins, may not have a profound influence on the clinical outcome of resected NSCLCs. Multiple factors regulating the G1/S transition, including cyclins D1 and E, cdk4 and cdk6, p27/KIP1 and p53 proteins as well as p16 and Rb proteins, may be involved in the clinical progression and outcome of NSCLCs in a complex manner (Quinlan et al, 1992; Horio et al, 1993; Mitsudomi et al, 1993; Xu et al, 1994, 1996; Fujino et al, 1995; Kelley et al, 1995; Kratzke et al, 1996; Nishio et al, 1996, 1997; Dosaka-Akita et al, 1997; Taga et al, 1997; Yatabe et al, 1998). It has been considered that NSCLCs are very heterogeneous both biologically and clinically because: (1) genetic alterations, including those of tumour suppressor genes and oncogenes, in NSCLCs are relatively diverse, and (2) each alteration is less frequent and specific to NSCLCs compared to genetic alterations in other types of cancer (Minna et al, 1997). Hence, molecular prognostic markers have not clearly emerged for NSCLCs yet (Moore and Lee, 1996). However, determination of alterations of multiple factors involved in more than one cell cycle regulatory and tumour suppressive pathway may provide increased information regarding survival and prognosis (Dosaka-Akita et al, 1997).

In conclusion, the presence or absence of expression of p16, Rb and p16/Rb proteins did not predict the survival and prognosis in the present cohort of patients with potentially curatively resected NSCLCs. However, further studies are needed to determine the prognostic importance of the loss of p16 and p16/Rb proteins in combination with other factors regulating the G1/S transition.

### REFERENCES

Beahrs OH, Henson DE, Hutter RVP and Kennedy BJ (1992) Lung. In: *Manual for Staging of Cancer*. 4th edn, pp. 115–122. American Joint Committee on Cancer: Chicago

Betticher DC, White GRM, Liu X, Kappeler A, Altermatt HJ, Thatcher N and Heighway J (1997) G1 control gene status is frequently altered in resectable non-small-cell lung cancer. *Int J Cancer (Pred Oncol)* **74**: 556–562

Cairns P, Mao L, Merlo A, Lee DJ, Schwab D, Eby Y, Tokino K, van der Riet P, Blaugrund JE and Sidransky D (1994) Rates of *p16* (*MTS1*) mutations in primary tumors with 9p loss. *Science* **265**: 415–417

Dosaka-Akita H, Shindoh M, Fujino M, Kinoshita I, Akie K, Katoh M and Kawakami Y (1994) Abnormal *p53* expression in human lung cancer is associated with histologic subtypes and patient smoking history. *Am J Clin Pathol* **102**: 660–664

Dosaka-Akita H, Hu SX, Fujino M, Harada M, Kinoshita I, Xu HJ, Kuzumaki N, Kawakami Y and Benedict WF (1997) Altered retinoblastoma protein expression in nonsmall cell lung cancer. *Cancer* **79**: 1329–1337

Fujino M, Dosaka-Akita H, Harada M, Hiroumi H, Kinoshita I, Akie K and Kawakami Y (1995) Prognostic significance of p53 and ras p21 expression in nonsmall cell lung cancer. *Cancer* **76**: 2457–2463

Gehan EA (1965) A generalized Wilcoxon test for comparing arbitrarily singly-censored samples. *Biometrika* **52**: 203–223

Geradts J, Kratzke RA, Niehans GA and Lincoln CE (1995) Immunohistochemical detection of the cyclin-dependent kinase inhibitor 2/multiple tumor suppressor gene 1 (*CDKN2/MTS1*) product $p16^{INK4A}$ in archival human solid tumors: correlation with retinoblastoma protein expression. *Cancer Res* **55**: 6006–6011

Hannon GJ and Beach D (1994) $p15^{INK4B}$ is a potential effector of TGF-β-induced cell cycle arrest. *Nature (Lond)* **371**: 257–261

Harrell FE Jr (1986) The PHGLM procedure. In: *Supplementary Library User's Guide*, Hastings RP (ed.), pp. 437–466. SAS Institute: Cary, NC

Hayashi N, Sugimoto Y, Tsuchiya E, Ogawa M and Nakamura Y (1994) Somatic mutations of the *MTS* (multiple tumor suppressor) 1/*CDK4I* (cyclin-dependent kinase-4 inhibitor) gene in human primary non-small-cell lung carcinomas. *Biochem Biophys Res Commun* **202**: 1426–1430

© 1999 Cancer Research Campaign

Horio Y, Takahashi T, Kuroishi T, Hibi K, Suyama M, Niimi T, Shimokata K, Yamakawa K, Nakamura Y, Ueda R and Takahashi T (1993) Prognostic significance of *p53* mutations and 3p deletions in primary resected non-small cell lung cancer. *Cancer Res* **53**: 1–4

Kaplan EL and Meier P (1958) Nonparametric estimation for incomplete observations. *J Am Stat Assoc* **53**: 457–481

Kelley MJ, Nakagawa K, Steinberg SM, Mulshine JL, Kamb A and Johnson BE (1995) Differential inactivation of CDKN2 and Rb protein in non-small-cell and small-cell lung cancer cell lines. *J Natl Cancer Inst* **87**: 756–761

Kinoshita I, Dosaka-Akita H, Mishina T, Akie K, Nishi M, Hiroumi H, Hommura F and Kawakami Y (1996) Altered $p16^{INK4}$ and retinoblastoma protein status in non-small cell lung cancer: potential synergistic effect with altered p53 protein on proliferative activity. *Cancer Res* **56**: 5557–5562

Kratzke RA, Greatens TM, Rubins JB, Maddaus MA, Niewoehner DE, Niehans GA and Geradts J (1996) Rb and $p16^{INK4a}$ expression in resected non-small cell lung tumors. *Cancer Res* **56**: 3415–3420

Lukas J, Parry D, Aagaard L, Mann DJ, Bartkova J, Strauss M, Peters G and Bartek J (1995) Retinoblastoma protein-dependent cell-cycle inhibition by the tumour suppressor p16. *Nature (Lond)* **375**: 503–506

Minna JD, Sekido Y, Fong KM, Gazdar AF (1997) Molecular biology of lung cancer. In: *Cancer: Principles & Practice of Oncology*, 5th edn, DeVita VT Jr, Hellman S and Steven A (eds), pp. 849–857. Lippincott: Philadelphia

Mitsudomi T, Oyama T, Kusano T, Osaki T, Nakanishi R and Shirakusa T (1993) Mutations of the p53 gene as a predictor of poor prognosis in patients with non-small-cell lung cancer. *J Natl Cancer Inst* **85**: 2018–2023

Moore DF Jr and Lee JS (1996) Staging and prognostic factors: non-small cell lung cancer. In: *Lung Cancer: Principles and Practice*, Pass HI, Mitchell JB, Johnson DH and Turrisi AT (eds), pp. 481–494. Lippincott: Philadelphia

Nakagawa K, Conrad NK, Williams JP, Johnson BE and Kelley MJ (1995) Mechanism of inactivation of *CDKN2* and *MTS2* in non-small-cell lung cancer and association with advanced stage. *Oncogene* **11**: 1843–1851

Nishio M, Koshikawa T, Kuroishi T, Suyama M, Uchida K, Takagi Y, Washimi O, Sugiura T, Ariyoshi Y, Takahashi T, Ueda R and Takahashi T (1996) Prognostic significance of abnormal p53 accumulation in primary, resected non-small-cell lung cancers. *J Clin Oncol* **14**: 497–502

Nishio M, Koshikawa T, Yatabe Y, Kuroishi T, Suyama M, Nagatake M, Sugiura T, Ariyoshi Y, Mitsudomi T, Takahashi T and Takahashi T (1997) Prognostic significance of cyclin D1 and retinoblastoma expression in combination with p53 abnormalities in primary, resected non-small-cell lung cancers. *Clin Cancer Res* **3**: 1051–1058

Okamoto A, Demetrick DJ, Spillare EA, Hagiwara K, Hussain SP, Bennett WP, Forrester K, Gerwin B, Serrano M, Beach DH and Harris CC (1994) Mutations and altered expression of $p16^{INK4}$ in human cancer. *Proc Natl Acad Sci USA* **91**: 11045–11049

Okamoto A, Hussain SP, Hagiwara K, Spillare EA, Rusin MR, Demetrick DJ, Serrano M, Hannon GJ, Shiseki M, Zariwala M, Xiong Y, Beach DH, Yokota J and Harris CC (1995) Mutations in the $p16^{INK4}$*/MTS1/CDKN2*, $p15^{INK4B}$*/MTS2*, and *p18* genes in primary and metastatic lung cancer. *Cancer Res* **55**: 1448–1451

Otterson GA, Kratzke RA, Coxon A, Kim YW and Kaye FJ (1994) Absence of $p16^{INK4}$ protein is restricted to the subset of lung cancer lines that retains wildtype RB. *Oncogene* **9**: 3375–3378

Otterson GA, Khleif SN, Chen W, Coxon AB and Kaye FJ (1995) *CDKN2* gene silencing in lung cancer by DNA hypermethylation and kinetics of $p16^{INK4}$ protein induction by 5-aza 2´deoxycytidine. *Oncogene* **11**: 1211–1216

Quinlan DC, Davidson AG, Summers CL, Warden HE and Doshi HM (1992) Accumulation of p53 protein correlates with a poor prognosis in human lung cancer. *Cancer Res* **52**: 4828–4831

Serrano M, Hannon GJ and Beach D (1993) A new regulatory motif in cell-cycle control causing specific inhibition of cyclin D/CDK4. *Nature (Lond)* **366**: 704–707

Shapiro GI, Edwards CD, Kobzik L, Godleski J, Richards W, Sugarbaker DJ and Rollins BJ (1995) Reciprocal Rb inactivation and $p16^{INK4}$ expression in primary lung cancers and cell lines. *Cancer Res* **55**: 505–509

Taga S, Osaki T, Ohgami A, Imoto H, Yoshimatsu T, Yoshino I, Yano K, Nakanishi R, Ichiyoshi Y and Yasumoto K (1997) Prognostic value of the immunohistochemical detection of $p16^{INK4}$ expression in nonsmall cell lung carcinoma. *Cancer* **80**: 389–395

Tam SW, Shay JW and Pagano M (1994) Differential expression and cell cycle regulation of the cyclin-dependent kinase 4 inhibitor $p16^{Ink4}$. *Cancer Res* **54**: 5816–5820

WHO (1982) *Histological Typing of Lung Tumours*, 2nd edition. *Am J Clin Pathol* **77**: 123–136

Xu HJ, Cagle PT, Hu SX, Li J and Benedict WF (1996) Altered retinoblastoma and p53 protein status in non-small-cell carcinoma of the lung: potential synergistic effects on prognosis. *Clin Cancer Res* **2**: 1169–1176

Xu H, Hu S and Benedict WF (1991*a*) Lack of nuclear RB protein staining in G0/middle G1 cells: correlation to changes in total RB protein level. *Oncogene* **6**: 1139–1146

Xu HJ, Hu SX, Cagle PT, Moore GE and Benedict WF (1991*b*) Absence of retinoblastoma protein expression in primary non-small cell lung carcinomas. *Cancer Res* **51**: 2735–2739

Xu HJ, Quinlan DC, Davidson AG, Hu SX, Summers CL, Li J and Benedict WF (1994) Altered retinoblastoma protein expression and prognosis in early-stage non-small-cell lung carcinoma. *J Natl Cancer Inst* **86**: 695–699

Yatabe Y, Masuda A, Koshikawa T, Nakamura S, Kuroishi T, Osada H, Takahashi T, Mitsudomi T and Takahashi T (1998) $p27^{KIP1}$ in human lung cancers: differential changes in small cell and non-small cell carcinomas. *Cancer Res* **58**: 1042–1047

Zhang X, Xu HJ, Murakami Y, Sachse R, Yashima K, Hirohashi S, Hu SX, Benedict WF and Sekiya T (1994) Deletions of chromosome 13q, mutations in *retinoblastoma 1*, and retinoblastoma protein state in human hepatocellular carcinoma. *Cancer Res* **54**: 4177–4182

*© 1999 Cancer Research Campaign* 
